# HPV Meets APOBEC: New Players in Head and Neck Cancer

**DOI:** 10.3390/ijms22031402

**Published:** 2021-01-30

**Authors:** Giuseppe Riva, Camilla Albano, Francesca Gugliesi, Selina Pasquero, Sergio Fernando Castillo Pacheco, Giancarlo Pecorari, Santo Landolfo, Matteo Biolatti, Valentina Dell’Oste

**Affiliations:** 1 Otorhinolaryngology Division, Department of Surgical Sciences, University of Turin, 10126 Turin, Italy; giuseppe.riva@unito.it (G.R.); giancarlo.pecorari@unito.it (G.P.); 2 Department of Public Health and Pediatric Sciences, University of Turin, 10126 Turin, Italy; camilla.albano@unito.it (C.A.); francesca.gugliesi@unito.it (F.G.); selina.pasquero@unito.it (S.P.); sergiofernando.castillopachecho@unito.it (S.F.C.P.); santo.landolfo@unito.it (S.L.)

**Keywords:** human papillomavirus, head and neck cancer, APOBEC, cytidine deaminase, squamous cell carcinoma

## Abstract

Besides smoking and alcohol, human papillomavirus (HPV) is a factor promoting head and neck squamous cell carcinoma (HNSCC). In some human tumors, including HNSCC, a number of mutations are caused by aberrantly activated DNA-modifying enzymes, such as the apolipoprotein B mRNA editing enzyme catalytic polypeptide-like (APOBEC) family of cytidine deaminases. As the enzymatic activity of APOBEC proteins contributes to the innate immune response to viruses, including HPV, the role of APOBEC proteins in HPV-driven head and neck carcinogenesis has recently gained increasing attention. Ongoing research efforts take the cue from two key observations: (1) APOBEC expression depends on HPV infection status in HNSCC; and (2) APOBEC activity plays a major role in HPV-positive HNSCC mutagenesis. This review focuses on recent advances on the role of APOBEC proteins in HPV-positive vs. HPV-negative HNSCC.

## 1. Introduction

Head and neck squamous cell carcinoma (HNSCC) include about 6% of all cases of tumors worldwide, representing the sixth most prevalent type of cancer. Its incidence is of 15.2 and 4.6 per 100,000 people in men and women, respectively [1].

Alcohol and tobacco abuse are the two main risk factors for HNSCC and seem to act in a synergistic fashion. However, in recent years, human papillomavirus (HPV) has emerged as an independent biological risk factor for the development of HNSCC. Indeed, in men younger than 50 years without a prior history of tobacco and alcohol consumption, HPV infection is directly associated with increased incidence of oropharyngeal tumors [2]. HPV-driven tumorigenesis appears to be particularly relevant in the oropharynx, where the base of the tongue and the tonsils are the most vulnerable sites. Furthermore, patients affected by HPV-related HNSCC have a different mutational profile and a better survival [3,4]. In particular, the discover of different mutational profiles in HPV-negative (HPV^−^) vs. HPV-positive (HPV^+^) HNSCC has raised the hypothesis that HPV infection may also play a role in gene regulation [3].

In certain human tumors, aberrantly activated DNA-modifying enzymes, such as the apolipoprotein B mRNA editing enzyme catalytic polypeptide-like (APOBEC) family of cytidine deaminases, have been linked to several DNA mutations [5,6,7,8]. Interestingly, APOBEC activation is also a well-established pathway in the innate immune response to viruses, including retroviruses, parvoviruses, hepatitis B virus (HBV), BK polyomaviruses, herpes simplex virus 1 (HSV-1), human cytomegalovirus (HCMV), Epstein–Barr virus (EBV), and HPV [9,10]. Indeed, APOBEC3A (A3A) and APOBEC3C (A3C) can hypermutate the HPV-16 genome, hampering its infectivity [9]. In addition, APOBEC3B (A3B) has been involved in HPV^+^ HNSCC mutagenesis [11]. Intriguingly, diminished exposure to exogenous carcinogens tends to favor APOBEC-mediated mutations of HPV^+^ HNSCC, whereas HPV^-^ HNSCC mostly retains a carcinogen-associated mutational profile [11,12,13].

This review will summarize and analyze our current understanding of the emerging role of APOBEC proteins in HNSCC pathogenesis, focusing on the mutagenic activity of this family of deaminases.

## 2. Head and Neck Squamous Cell Carcinoma

HNSCC is a highly heterogeneous tumor typically arising from the epithelial cells of the mucosa, with the oral cavity, oropharynx, hypopharynx, and larynx being the most frequently affected sites [2,3]. Besides smoking and alcohol abuse, high-risk HPV infection is the main biological risk factor of HNSCC development in the oropharyngeal area, including 22–47% of oropharyngeal HNSCCs [14], with approximately 90% of HPV-related oropharyngeal carcinomas being caused by HPV-16 [15]. Thus, depending on their HPV infection status, HNSCCs can either be HPV^+^ or HPV^-^, with each group characterized by specific clinical manifestations, incidence patterns, molecular signatures, and prognoses [3,16,17,18,19]. For instance, HPV^+^ HNSCC has a tendency to present as a small primary tumor with large nodal metastases [20]. Furthermore, the incidence of HPV^+^ HNSCC in non-smoking and non-drinking younger subjects is much higher than that of HPV^-^ HNSCC. With regard to differences in their molecular profiles, while a smoking-associated mutational signature was detected in a sample of HPV^-^ HNSCCs, reduced exposure to exogenous carcinogens in HPV^+^ HNSCC patients correlated with an APOBEC mutational burden [11,12]. In terms of differences in overall survival, HPV^+^ HNSCCs generally display a better response to chemoradiotherapy [21] and a more favorable prognosis [20].

The highest prevalence of HPV infection in HNSCC has been reported in South America and central, eastern, and northern Europe, whereas southern European regions tend to have much lower incidence rates [14]. Moreover, in the past decades, the proportion of HPV-related oropharyngeal tumors has increased approximately 4-fold—from 7.2% in 1990–1994 to 32.7% in 2010–2012—seemingly as a result of altered sexual behaviors, as HPV^+^ tumors are linked to oral sex [22].

Non-oropharyngeal HNSCCs i.e., tumor affecting the oral cavity, larynx and hypopharynx, are less frequently related to HPV infection, with HPV DNA prevalence in oral and laryngeal tumors being 23.5% and 24.0%, respectively [15]. It is also interesting to point out that not all HPV^+^ tumors are positive for HPV E6 and E7 gene expression, two viral oncogenes considered to be the most reliable biomarkers of HPV-mediated transformation [23]. For instance, HPV DNA and E6/E7 mRNA could only be detected in 21.8%, 3.9%, and 3.1% of oropharyngeal, oral, and laryngeal tumors, respectively [14].

HPV proteins E6 and E7 play a functional role in cancer development through inactivation of p53 and retinoblastoma protein (pRb) [23,24]. In particular, pRb loss upregulates p16 protein expression, which in turn shuts down cyclin D1/cyclin-dependent kinase 4/6 (CDK4/6) signaling, thereby blocking the G1/S transition. The fact that p16 is now widely used as a molecular marker of HPV infection has led to the recent modification of the oropharyngeal Tumor Node Metastasis (TNM) staging system so as to include p16 positivity among the criteria for predicting HPV status [20]. However, the observation that p16 overexpression does not always coincide with HPV DNA positivity has questioned the use of this protein as a marker of HPV infection in HNSCC [25,26].

As aforementioned, HPV positivity and negativity determine distinctive molecular signatures among HNSCCs. The following sections summarize our current knowledge of this clinically relevant phenomenon.

The majority of HPV^-^ HNSCCs harbor loss of chromosomes 3p and 9p as well as tumor protein 53 (TP53) mutations [27]. In such tumors, the amplification of cyclin D1 and the loss of the tumor suppressor gene CDKN2A (p16INK4A) drive neoplastic cells through the G1/S checkpoint, contributing to DNA replication [28]. Moreover, p53-mediated cell cycle arrest and apoptosis in response to DNA damage is defective in these tumors because TP53 is usually inactivated by missense mutations and allelic loss [27]. Fittingly, TP53 somatic mutations, present in 30–75% of HNSCCs, correlate with poor survival of patients with invasive carcinomas [29,30].

Approximately 15% of HPV^-^ HNSCCs harbor NOTCH1 mutations, which make this transmembrane receptor the second most frequently mutated gene in HNSCC [4,16]. NOTCH1 regulates normal cell differentiation, lineage commitment, and embryonic development, and besides acting as a transcriptional activator can function as a tumor suppressor [16,31]. Another gene structurally similar to NOTCH1 that is instead amplified in HPV^-^ HNSCC is the epidermal growth factor receptor (EGFR), which plays a well-established role in cell proliferation. Lastly, HPV^-^ HNSCCs often display amplification of the MET proto-oncogene receptor tyrosine kinase, which drives migration, invasion and angiogenesis of cancer cells when constitutively activated [32,33].

On the other hand, HPV^+^ HNSCCs contain a lower number of mutations per tumor in comparison with HPV^-^ HNSCCs and hardly ever display inactivating p16INK4A mutations [16,31,34,35]. Furthermore, most of those genes that are aberrantly expressed in HPV^-^ HNSCCs are usually unaffected in their HPV^+^ counterparts, as shown by several epigenetic and genomic studies [19]. For example, HPV^+^ HNSCCs generally express the wild-type conformation of the tumor suppressor gene TP53 [19].

Another difference between HPV^-^ and HPV^+^ HNSCCs lies in expression of the two PYHIN proteins IFI16 and AIM2, the former nuclear and the second cytoplasmic sensors of double strand (ds) DNA. More specifically, while IFI16 is generally upregulated in HPV^+^ HNSCC, AIM2 gene expression levels are usually found unchanged in HPV^+^ HNSCCs with respect to their HPV^-^ counterparts, where this gene is already expressed at high levels (10% vs. 50% of cases, respectively) [12,13].

Lastly, while HPV^+^ HNSCCs carry mutations in the helical domain of the PIK3CA gene, HPV^-^ HNSCC harbors mutations across the entire gene. PIK3CA encodes the catalytic subunit of phosphoinositide 3-kinase (PI3K) p110α, which triggers AKT signaling. PIK3CA amplification, and/or mutations have been found in 34% of HPV^-^ and 56% of HPV^+^ HNSCCs [16]. The recent observation that the helical domain of the PIK3CA gene is subject to APOBEC-induced mutations in multiple cancers has led to the hypothesis that APOBEC activity may play a key role in HPV-induced transformation [11]. This assumption is also supported by the fact that in HPV-related cancers induction of APOBEC mutagenesis may also be the result of gene amplification besides viral infection [6]. In this regard, the role of APOBEC3 family of cytidine deaminases has been recently demonstrated in HPV^+^ HNSCCs [12], in good agreement with previous findings showing how the mutational signature of APOBEC can determine a specific mutational profile in these tumors [36].

The role of APOBEC-mediated cytosine deamination in generating driver mutations is discussed in more detail below.

## 3. The APOBEC Family and Cytosine Deamination

AID (activation-induced deaminase)/APOBEC is a family of enzymes with cytidine deaminase activity capable of converting cytosines into uracils in either RNA and/or single strand (ss) DNA, thus inducing missense mutations or early stop codons.

The human AID/APOBEC protein family comprises the following 11 members: Activation-induced cytidine deaminases (AIDs) and APOBEC1 (A1) (genes located on chromosome 12), APOBEC2 (A2) (chromosome 6), 7 APOBEC3 (A3) proteins (i.e., A3A, A3B, A3C, A3D, A3F, A3G, and A3H) (chromosome 22), and APOBEC4 (A4) (chromosome 1) [8,37].

Each member of this family shares at least one zinc-dependent deaminase (ZDD) containing the consensus amino acid sequence H-X-E-X23-28-P-C-X2-4-C, where X represents any amino acid. While the histidine (H) and cysteine (C) residues bind zinc at the active site, the glutamic acid residue (E) regulates proton shuttling through deamination [37,38,39]. APOBECs catalyze cytosine deamination into uracil through a zinc-mediated hydrolytic mechanism. Specifically, the conserved glutamic acid in its zinc-binding deaminase motif mediates water deprotonation. This results in a zinc-stabilized hydroxide ion that promotes nucleophilic substitution of the amine group of the cytosine at position 4 with a carbonyl group. Each of the APOBEC proteins deaminates cytosines at specific consensus sequences, with the amino acid sequence at the 5′ of the target cytosine being unique to each APOBEC enzyme—i.e., 5′ WRCY for AID1 (W = A or T; R = Purine), 5′ AC for A1, 5′ CC for A3G and 5′ TC for all the other A3 enzymes (A3A, A3B, A3C, A3D, A3F, and A3H) [40,41].

**Figure 1:**
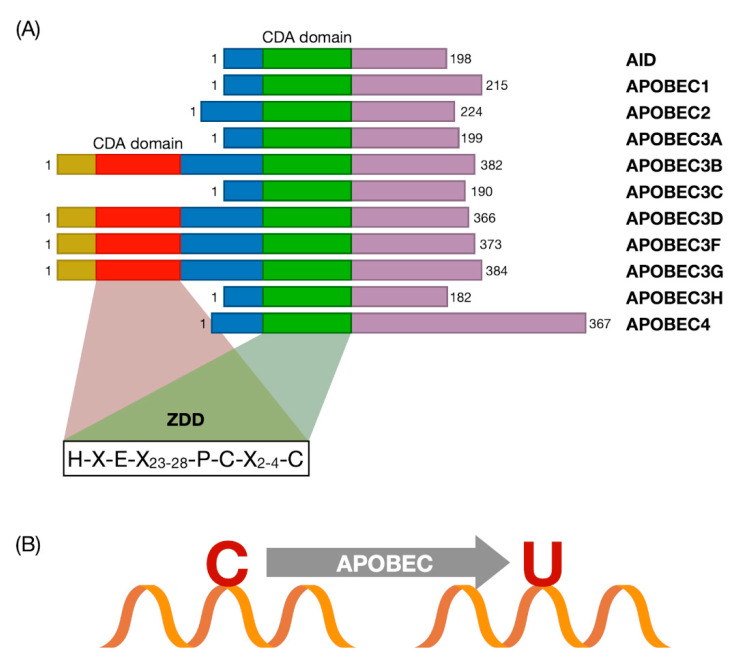
(**A**) Schematic representation of the apolipoprotein B mRNA editing enzyme catalytic polypeptide-like (APOBEC) family genes. The diagram shows the structural organization of APOBEC family proteins. The domains and position of the evolutionarily conserved residues are shown. The red box represents the catalytically inactive cytidine deaminase (CDA) domain, while the green box is the catalytically active CDA domain. The zinc-dependent deaminase (ZDD) consensus amino acid sequence (H-X-E-X23-28-P-C-X2-4-C) is shown. X represents any amino acid. Histidine (H) and cysteine (C) residues bind zinc at the active site, the glutamic acid residue (E) controls proton shuttling through deamination. CDA: Catalytic site cytidine deaminase. ZDD: Zinc-dependent deaminase. (Modified from [39]) (**B**) Reaction of the ssDNA cytosine deamination catalyzed by APOBEC family members.

APOBEC cytosine deamination activity is mediated by the catalytic site cytidine deaminase (CDA) domain containing a highly conserved ZDD sequence motif comprising five β-strands stabilized by six α-helices (Figure 1).

The substrate specificity and function of the catalytic activity of APOBEC are influenced by the variations in the length, composition, and spatial distribution of these highly conserved secondary structures. Specifically, the conformation of the loops in proximity of the CDA regulates both substrate recognition and catalytic site interaction with the substrate. For instance, loop 7 contains a conserved sequence that, by interacting with loops 1, 3, and 5, determines the sequence targeting specificity of all AID/APOBEC enzymes [42]. Another example is the interaction of ssDNA/RNA with APOBECs, which occurs thanks to the combined action of shallow grooves on the protein surface and the catalytic site rich in basic and hydrophobic residues capable of binding to negatively charged nucleic acids [41]. In addition, the catalytic activity of several APOBECs is determined by their ability to form high molecular weight homo and hetero complexes. For instance, APOBEC1 can heterodimerize with the A1 complementation factor (A1CF), an RNA binding protein co-factor, while AID is functionally active in both homodimer and monomer conformations. Interestingly, A3B, A3D, A3F, A3G, and A3H can only be detected as multimeric forms, whereas A2, A3A, and A3C can exclusively form monomers. Oligomers are then thought to influence the spatial orientation of the loops involved in ssDNA/RNA binding and catalytic site access, thereby regulating the deamination kinetics [43,44].

Each APOBEC family member exhibits different functions at the cellular level. For example, cytidine deamination by APOBEC proteins plays an important role in sensing endogenous and exogenous retroviruses and triggering innate and adaptive immunity. Furthermore, this catalytic activity is involved in the control of epigenetic mechanisms and lipid metabolism [45].

Another peculiar property of APOBEC is their ability to increase tumor mutations due to aberrant DNA editing [46,47,48,49]. APOBEC genes are quite often aberrantly regulated in cancer, which then leads to increased mutagenesis and genomic instability in cancer cells. Thus, there has been an increasing interest in determining whether altered expression profiles of APOBECs in tumor cells can increase the occurrence of somatic mutations. This may be in part supported by recent findings showing that germline variations of APOBECs are associated with increased mutagenic capacity, which then may promote tumorigenesis [39]. In this regard, whole exome and genome sequencing analyses have revealed the presence of an APOBEC mutation signature in multiple cancer types, including breast, bladder, thyroid, and cervix cancer, B cell lymphoma, lung adenocarcinoma (LUAD), acute lymphoblastic leukemia (ALL) and chronic lymphocytic leukemia (CLL), and multiple myelomas (MM). By contrast, other cancers, such as acute myeloid leukemia (AML) and colorectal and liver cancer, do not seem to contain any APOBEC signatures [50].

More recently, Burns et al. have reported high APOBEC3B mRNA levels in most of primary breast tumors and breast cancer cell lines examined. Of note, A3B expression and catalytic activity were associated with increased genomic uracil levels, dC > dT transition rates and mutation frequencies [51]. Finally, APOBEC3 proteins have been proposed to link viral infections to cancer development [39], an interesting aspect that will be further addressed in the following section.

## 4. APOBEC Genes vs. HPVs: Restriction Factors or DNA Editors?

Restriction factors (RFs) are the first line of defense against different viruses, including HPVs [52,53]. Among RFs, A3 genes have emerged as an integral part of intrinsic immunity: they are potently induced by interferons following viral infection and exert antiviral activities through deamination-independent and -dependent mechanisms [54], which has led many to hypothesize that an abnormal regulation of this response may trigger somatic mutagenesis in cancer cells.

APOBEC3 proteins were initially discovered for their activity against retroviruses [55], but later on, it became evident that they were also effective against a wide range of other viruses, such as parvoviruses [56], HBV [57,58], BK polyomaviruses [59,60], HSV-1 [61], HCMV [62], and EBV [63]. In this regard, our group has recently uncovered a novel mechanism of HCMV evasion from A3 restriction activity based on the progressive loss of APOBEC hot spots from its genome during evolution [10]. A3 inactivation as a means to evade host immune surveillance does not seem to be solely restricted to HCMV as other viruses, such as HSV-1 and Kaposi’s sarcoma-associated herpesvirus (KSHV), can delocalize A3 proteins and, in doing so, neutralize their antiviral activity through a conserved ribonucleotide reductase (RNR)-dependent mechanism [64]. More recently, APOBECs have been involved in SARS-CoV-2 RNA editing [65]. Finally, a close relationship between APOBEC and HPV is suggested by the fact that APOBEC-driven mutations are frequent in cervical cancer, and that APOBEC expression is upregulated in HPV-infected cells [11]. The first hint that A3s were restriction factors for HPVs came from in vitro studies showing that HPV pseudovirions, once packaged in 293T overexpressing A3A or A3C, displayed reduced infectivity in keratinocytes, an effect that could be reversed by A3A silencing [9,66,67]. An additional piece of evidence supporting the role of A3 proteins in HPV restriction was brought by experiments showing that catalytically inactive A3A mutants were unable to curb HPV infectivity in keratinocytes [68]. Importantly, the fact that HPV pseudovirion genomes isolated from A3A-overexpressing cells lack A3 RNA-editing activity further supports a mechanism by which A3 acts as a restriction factor for HPV in a deaminase-dependent fashion [68,69].

Among A3 genes, A3A is the more involved in HPV regulation, whereas A3B seems to have no effect on HPV infection [68]. More specifically, A3A expression negatively correlates with the number of encapsidated pseudovirions [68]. Interestingly, A3A, and also A3C, inhibit viral entry by binding to the HPV L1 capsid protein, suggesting that A3 species acts at different stages of the viral cycle [9].

A physiological process where the interplay between HPV and A3 is even more evident is the cell cycle. While HPV stimulates cell cycle progression through the S-phase, ectopic expression of A3A induces cell cycle arrest, probably as a result of the DNA damage triggered by its cytidine deaminase activity [70,71,72]. Thus, it seems that A3 can act in two different ways to restrict HPV replication: (i) Directly, by attacking the virus or (ii) indirectly, by inducing cell-cycle arrest. A direct effect of A3 on the HPV genome has been widely demonstrated in both in vitro and in vivo models. In particular, following A3A overexpression, HPV E2 appears to be one of main targets of A3 in the HPV-16^+^ low-grade CIN (cervical intraepithelial neoplasia) lesion-derived cell line W12 [73] as well as in precancerous cervical lesions [74]. An enrichment of A3 editing events has also been observed in the LCR region of HPV-1^+^ warts and HPV-16^+^ precancerous cervical lesions [75,76]. Among A3 transcripts, A3A and A3C were prevalent, while A3B was expressed at a much lower levels in HPV-infected cervix. Given that A3-driven mutations are very frequently detected in CIN1 lesions, it is possible that A3 editing may preferentially occur during stages of productive infection, when HPV virions are being released [77].

In general, the frequency of HPV editing is significantly lower compared to other viruses, such as human immunodeficiency virus 1 (HIV-1) or HBV, which makes deamination an unlikely event during HPV restriction. This would not, however, preclude an involvement of A3 activity in driving HPV evolution through the introduction of variations that would otherwise be lacking in a DNA virus (e.g., HPV) that is duplicated with a much higher fidelity compared to any RNA virus. In this regard, the selective pressure exerted by A3-mediated editing has been proposed as the potential cause of TC dinucleotide depletion in high-risk α-HPV genomes [66]. On the other hand, A3A expression levels are significantly higher in mucosal tissue, the main target of α-HPV, compared to cutaneous skin, where β-HPVs are predominant [66]. Although such mutations may be deleterious for the virus, when they do not affect viral fitness, they might favor viral evasion from immune surveillance, thereby allowing positive selection of viral strains within the host. Thus, the HPV sequences present in cancer cells should not just be regarded as the result of A3 editing activity but also the consequence of the selective pressure against loss/enhancement of host cell fitness. Indeed, sequencing of HPV-16 genomes from 5570 samples representing productive, precancerous, and invasive lesions displayed a remarkable degree of inter-host variation that was more enhanced in productive lesions [78]. 

**Figure 2:**
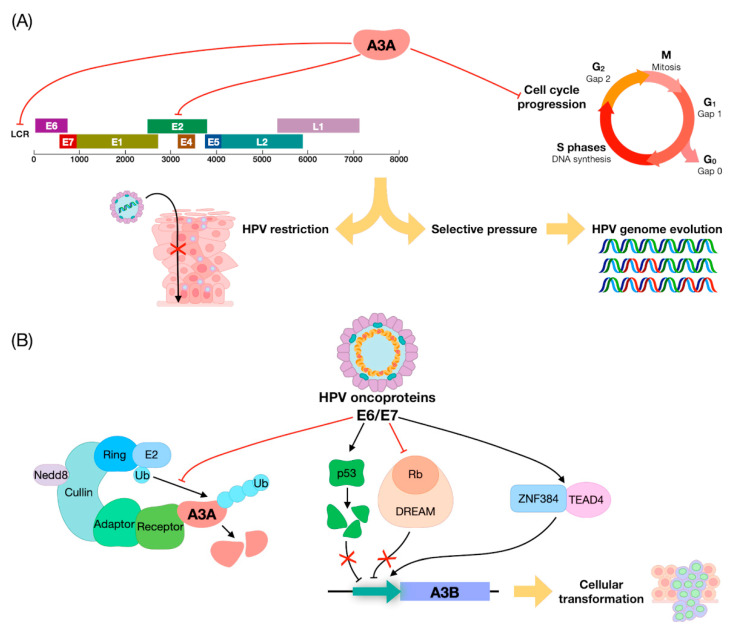
Summary of APOBEC3/HPV interplay. (**A**) Negative regulation exerted by A3A on human papillomavirus (HPV) gene expression and HPV-driven cell-cycle progression, leading to HPV restriction and genome evolution. (**B**) A3 modulation by HPV oncoproteins E6/E7: (i) cullin-RING-based E3 ubiquitin ligase-mediated polyubiquitination followed by A3A degradation; (ii) A3B upregulation through removal of the inhibitory activity exerted by p53 and Rb or through a direct effect of the zinc finger protein ZNF384 and TEAD4 on A3B promoter.

Another important aspect that should be considered is how HPVs regulate A3 genes. The first evidence of the existence of such mechanism came from the observation that A3B expression was upregulated by E6/E7 in keratinocytes in vitro [79]. Importantly, E7 from high-risk HPV types was later found to inhibit cullin-RING-based E3 ubiquitin ligase-mediated polyubiquitination and degradation of A3A. Furthermore, A3 genes, especially A3B, were found to be upregulated in precancerous cervical lesions, in good agreement with the aforementioned transactivation by E7 in keratinocytes [68,80,81]. Moreover, A3B upregulation relied on the presence of E6-responsive regions in the A3B promoter, which in human keratinocytes made it inducible by E6 through the zinc finger protein ZNF384 and the TEA Domain Transcription Factor 4 (TEAD4) [82,83]. An additional piece to this complicated puzzle has been recently added by findings showing that both E6 and E7 from HPV-16 can independently upregulate A3B expression in immortalized keratinocytes, albeit through different mechanisms. E6 degrades p53, thus removing p53 repression of A3B [84], while E7 binds to pRB family members (pRB, p107, and p130) for degradation [85] (Figure 2).

Overall, HPV-mediated upregulation of A3 genes may be linked to the evolutionary advantage that the virus would gain by inducing a certain level of A3 expression. In this regard, it is important to highlight that A3B, unlike A3A, does not inhibit HPV infectivity [68], indicating that the function of A3 in HPV restriction is entirely separate from its editing activity during tumor development, with the former mediated mainly by A3A and/or A3C and A3H, and the latter by A3B.

## 5. The Role of APOBEC in HNSCC

The hypothesis that APOBEC-driven mutagenesis is linked to cancer development derives from The Cancer Genome Atlas (TCGA) genome analyses showing that 68% of all mutations in bladder, cervical, breast, head and neck, and lung tumors are consistent with an APOBEC mutation pattern, and target cancer driver genes [6].

The functional role of APOBEC in HNSCC pathogenesis is supported by the following lines of evidence, summarized in Table 1.

Among HNSCCs, a higher APOBEC activity has been observed in HPV^+^ HNSCCs [11,12,86,87]. In particular, while 98% of HPV^+^ HNSCCs exhibit an APOBEC signature, this latter is only present in 76% of HPV^-^ HNSCCs [86]. Indeed, as mentioned in Section 2 of this review, HPV^-^ HNSCCs display a smoking-associated mutational signature, whereas HPV^+^ HNSCCs from patients less exposed to external carcinogens harbor a mutation pattern consistent with APOBEC DNA editing. Moreover, as previously discussed, APOBEC activity has been involved in the generation of helical domain hot spot mutations in the PIK3CA gene in HPV^+^ HNSCC [11].

Another important finding attesting the role of APOBEC in HNSCC development is that APOBEC enrichment directly correlates with the overall mutational burden of HPV^+^ HNSCC [7,88]. In particular, bioinformatics analysis of tumor exomes, transcriptomes, and germline exomes from 511 HNSCC patients from TCGA revealed a striking correlation between A3A expression and APOBEC mutation rate [7]. Consistently, enhanced expression of A3A was strongly correlated with the rate of HPV integration in oropharyngeal cancer biopsies [89]. Lastly, the observation that HPV16^+^ tumors have a higher proportion of APOBEC-related mutations compared to HPV33^+^ tumors (57% vs. 23%) suggests that the extent of APOBEC mutagenesis depends on the HPV serotype [90].

More evidence supporting a pro-tumorigenic role of APOBEC comes from immunohistochemistry and reverse transcription quantitative PCR analyses reporting higher expression levels of A3A and A3B in HPV^+^ vs. HPV^-^ HNSCCs [12,87,89], with only one immunofluorescence-based study failing to detect enhanced A3 protein expression in HPV^+^ oropharyngeal cancers [91]. In particular, RT-qPCR of samples from tumor tissues and healthy mucosa showed low mRNA expression levels of A3A and A3B in HPV^-^ HNSCC, whereas their expression was upregulated by 2–100 folds in HPV^+^ cancers [12]. Similar to A3A, A3H was found to be predominantly upregulated in HPV^+^ HNSCC [92].

Although A3A and A3B appear to be mainly overexpressed in HPV^+^ cancer, they have also been found to be upregulated in some subsets of HPV^-^ HNSCCs [93,94,95,96]. In addition, the fact that low-grade HPV^-^ oral dysplasia show intermediate levels of A3B protein expression, while high-grade oral dysplasia has much higher A3B protein levels, suggests that this gene may be progressively activated in HPV^-^ oral cancers. Furthermore, the strong positive correlation between nuclear A3B and Ki67 scores observed in these tumors suggests a role of this gene in cell proliferation [95].

Cellular localization and tumor stage also appear to be important determinants of A3 protein expression. Immunohistochemistry of oral squamous cell carcinomas, which are predominantly HPV^-^ tumors, has in fact revealed a more prominent cytoplasmic localization of A3B in a subgroup of tumor cells compared to normal oral epithelial cells, where A3B is mainly localized in the nucleus [96]. Besides A3B, also the expression pattern of AID changes according to tumor stage. In particular, AID expression is more frequently detected in early stage oral squamous cell carcinomas [97]. Moreover, a study on a Taiwanese sample reported a significantly higher expression of A3A in oral tumors carrying A3B-deletion alleles. The frequency of A3B-deletion germline polymorphism was found to be much higher in Taiwan in comparison with TCGA (50% vs. 5.8%). Although HPV status was not assessed, high levels of A3A expression were associated with better overall survival, especially among individuals carrying A3B-deletion alleles [93].

A correlation between APOBEC expression and other genes has been observed in RT-PCR-based investigations [12]. In HPV^-^ HNSCC, statistically significant positive correlations were found between: (i) TP53 expression and A3A and A3F expression; (ii) NOTCH1 expression and A3B and A3F expression; and (iii) PD-L1 expression and A3A expression. In HPV^+^ HNSCC, positive correlations were found between (a) MET expression and A3A expression; (b) PD-L1 expression and A3F expression; and (c) IFI16 expression and A3A expression. However, following false discovery rate (FDR) adjustment, only the correlation between PD-L1 expression and A3F expression remained significant [12]. Another interesting association is between A3A and estrogen receptor α (ERα) expression in HPV^+^ oropharyngeal squamous cell carcinomas. In particular, A3A mRNA is induced by estrogen in HPV^+^ ERα^+^ oropharyngeal carcinoma cells [98]. Lastly, head and neck tumors with high A3H levels exhibit a genome-wide DNA hypomethylation pattern [92].

More importantly, emerging evidence indicates that APOBEC-driven mutagenesis correlates with the initiation of several immune pathways in both HPV^+^ and HPV^-^ HNSCC [7,99]. In particular, the activation of CD8^+^ T cells was associated with APOBEC mutational burden, even when these cell populations were adjusted for HPV status. Moreover, overall T-cell populations along with eosinophil, dendritic, and cytotoxic cells and B-cell subsets correlated with APOBEC mutational burden. Interestingly, among these populations, cytotoxic T cells displayed the strongest correlation rate in HPV^-^ HNSCC but were not associated with age and smoking, two well-established mutational signatures in HNSCC (see Section 2) [99]. Globally, such results demonstrate that APOBEC has a role in immune response, not only in HPV^+^ HNSCC, but also in a subset of HPV^-^ tumors.

HPV^+^ HNSCC had the highest levels of IFN-γ, which is known to upregulate PD-L1 in tumors [100]. The fact that PD-L1 expression in HNSCC correlates with APOBEC mutations supports a potential role of APOBEC-dependent mutagenesis in immune checkpoint inhibition in cancer cells [12,100]. Indeed, tumor specific neoantigens (i.e., mutation-derived class I binding neoantigens), which correlates with response to immunotherapy, have been found to be significantly associated with APOBEC mutational burden, contrary to other sources of neoantigens [100]. Another important correlation found in HPV^+^ HNSCC involves A3H expression and activation of the immune response. Specifically, A3H was shown to demethylate and upregulate CXCL10, thereby enhancing CD8^+^ T cell tumor infiltration [92]. Lastly, an APOBEC-enriched subgroup with higher T-cell inflammation and immune checkpoint expression has been identified in HPV^-^ HNSCC [99]. Globally, these results suggest a role of APOBEC in the response to cancer immunotherapy.

Survival analyses have shown that some members of APOBEC family affec overall survival (OS) [12,92,101]. In HPV^+^ HNSCC, higher A3G or A3H expression correlates with better OS [92,101]. Moreover, APOBEC3s knockdown in HNSCC cells results in resistance to cisplatin and carboplatin as well as in increased rates of cisplatin-induced interstrand crosslink removal in HNSCC cells. These data support the hypothesis that APOBEC3s activate base excision repair in HNSCC, mediate repair of cisplatin-induced interstrand crosslinks, and consequently sensitize cells to cisplatin. Such effects contribute to the improved treatment responses observed in HPV^+^ HNSCC patients [101]. On the other hand, in HPV^-^ HNSCC, upregulation of A3F gene expression correlated with a worse prognosis, and patients displaying stable A3H expression levels had a lower OS [12]. The different impact of A3H expression on OS in HPV^+^ vs. HPV^-^ cancer patients may be related to the different mutational burden in these two tumor subgroups. Further studies are clearly needed to better understand this particular feature of A3H.

## 6. Conclusions and Future Perspectives

In conclusion, emerging evidence from recent biochemical and bioinformatic analyses supports a functional role of APOBEC family members in both HPV^+^ and HPV^-^ HNSCC pathogenesis. In particular, two of these genes, A3A and A3B, show distinct mutational signatures, with the former conferring a smoking-associated mutational signature to HPV^-^ cancers, and the latter mainly acting as generator of driver mutations in HPV^+^ HNSCC under reduced exposure to exogenous carcinogens. In addition to influencing the mutational burden of cancer cells, APOBEC-driven mutagenesis is being increasingly involved in cancer immune response, cisplatin sensibility, and overall survival. The key finding that emerges from this review is that HNSCC patients with higher APOBEC expression levels tend to have a better response to chemotherapy and immunotherapy, which, given the positive correlation between HPV and A3 gene expression, may help explain the better survival of HPV^+^ vs. HPV^-^ cancer patients. Further studies are thus urgently needed to conclusively determine whether A3 genes, especially A3H, are potential predictive biomarkers for cancer immunotherapy.

## Figures and Tables

**Table 1:** Main features and role of APOBEC in HPV^+^ and HPV^-^ head and neck squamous cell carcinoma (HNSCC).

HPV^+^	HPV^-^
Upregulation of A3A, A3B and A3H [12,87,89,92]. Positive correlation with mutational burden [7,88,90]. Generation of helical domain hot spot mutations in the PIK3CA gene [11]. Correlation between A3A expression and integration of HPV DNA [89]. Positive correlation between: PD-L1 and A3F expression [12,100]; A3A and Erα [98], IFI16 and MET [12] expression. Association between A3H expression levels and genome-wide DNA hypomethylation pattern [92]. Positive correlation between A3H and CD8^+^ T cell infiltration [92]. Higher A3G or A3H expression correlates with better overall survival [92,101].	Upregulation of A3A and A3B in some subsets of tumors [93,94,95,96]. Positive correlation between: A3A and A3F and TP53; A3B and A3F and TP53; A3A and PDL1 [12]; A3B and Ki67 [95]. Higher T cell inflammation and immune checkpoint expression in an APOBEC-enriched subgroup [99]. A3F upregulation correlates with a worse prognosis, whereas patients without changes in A3H expression have a lower overall survival [12].
